# Exogenous Cushing syndrome

**DOI:** 10.11604/pamj.2023.46.45.41228

**Published:** 2023-09-29

**Authors:** Ashwin Karnan, Anjana Ledwani

**Affiliations:** 1 Department of Respiratory Medicine, Jawaharlal Nehru Medical College, Datta Meghe Institute of Higher Education and Research, Sawangi (Meghe), Wardha, Maharashtra, India

**Keywords:** Cushing syndrome, steroids, cortisol

## Image in medicine

A 10-year-old girl presented to our hospital with acute respiratory distress and diminished vision for the past 10 days. She was a recently diagnosed case of diffuse midline glioma who was on palliative care and had been extensively treated with oral corticosteroids by a local physician. On examination, she was afebrile, had a pulse rate of 104 beats per minute, respiratory rate of 28 breaths per minute, and blood pressure of 150/70 mmHg, on auscultation, there were bilateral crepitations, had a round, moon-like face with acne and papular lesions, dorsocervical fat deposition, central obesity with marked striae. All routine investigations were done. She was stabilized and started on intravenous antibiotics. Twenty-four (24) hour urine-free cortisol level was high which was suggestive of Cushing syndrome. An ophthalmology evaluation was done, and she was diagnosed with central serous chorioretinopathy. The steroid was slowly tapered, and the patient was transferred to the oncology department for radiotherapy. Exogenous Cushing syndrome is common in clinical practice. The commonest signs and symptoms include moon facies, supraclavicular fat pads, buffalo hump, ecchymoses, facial plethora, skin thinning, hirsutism, acanthosis nigricans, central obesity, peptic ulcers, high blood pressure, proximal muscle weakness, osteoporosis, glaucoma, cataract and avascular necrosis of the hip. The treatment for exogenous Cushing syndrome is a gradual withdrawal of the drug. A patient with hypothalamic-pituitary-adrenal axis suppression cannot increase steroid production during illness or stress and hence should receive stress-dose steroids to prevent adrenal crisis.

**Figure 1:**
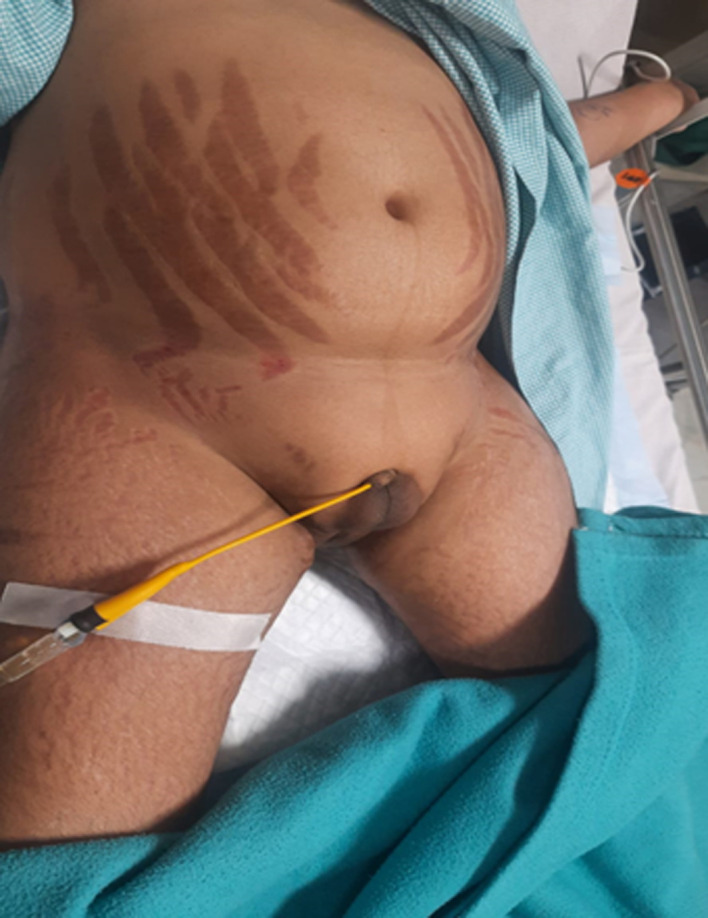
central obesity with marked striae

